# Specificity Re-evaluation of Oligonucleotide Probes for the Detection of Marine Picoplankton by Tyramide Signal Amplification-Fluorescent *In Situ* Hybridization

**DOI:** 10.3389/fmicb.2017.00854

**Published:** 2017-05-29

**Authors:** Virginie Riou, Marine Périot, Isabelle C. Biegala

**Affiliations:** Centre National de la Recherche Scientifique, Mediterranean Institute of Oceanography – Institut de Recherche pour le Développement, Aix Marseille Université – Université de ToulonMarseille, France

**Keywords:** TSA-FISH, probes, specificity, marine, prokaryotes, eukaryotes, oligonucleotide, CARD-FISH

## Abstract

Oligonucleotide probes are increasingly being used to characterize natural microbial assemblages by Tyramide Signal Amplification-Fluorescent *in situ* Hybridization (TSA-FISH, or CAtalysed Reporter Deposition CARD-FISH). In view of the fast-growing rRNA databases, we re-evaluated the *in silico* specificity of eleven bacterial and eukaryotic probes and competitor frequently used for the quantification of marine picoplankton. We performed tests on cell cultures to decrease the risk for non-specific hybridization, before they are used on environmental samples. The probes were confronted to recent databases and hybridization conditions were tested against target strains matching perfectly with the probes, and against the closest non-target strains presenting one to four mismatches. We increased the hybridization stringency from 55 to 65% formamide for the Eub338+EubII+EubIII probe mix to be specific to the *Eubacteria* domain. In addition, we found that recent changes in the *Gammaproteobacteria* classification decreased the specificity of Gam42a probe, and that the Roseo536R and Ros537 probes were not specific to, and missed part of the *Roseobacter* clade. Changes in stringency conditions were important for bacterial probes; these induced, respectively, a significant increase, in Eubacteria and *Roseobacter* and no significant changes in *Gammaproteobacteria* concentrations from the investigated natural environment. We confirmed the eukaryotic probes original conditions, and propose the Euk1209+NChlo01+Chlo02 probe mix to target the largest picoeukaryotic diversity. Experiences acquired through these investigations leads us to propose the use of seven steps protocol for complete FISH probe specificity check-up to improve data quality in environmental studies.

## Introduction

Marine prokaryotes and picoeukaryotes (0.2–3 μm) play major roles in biogeochemical cycles (Falkowski et al., 2008; Worden and Not, 2008). Their quantification is a pre-requisite to characterize their relative contribution to ecosystem functioning, and thus understand their role in the Earth’s global changes (Le Quéré et al., 2005) related to ocean acidification (Hutchins et al., 2009) or coastal eutrophication (Smith and Schindler, 2009). Species-specific monitoring techniques are also needed for the early detection and follow-up of harmful species that are increasing sources of nuisances to human and ecosystem health, such as *Vibrio vulnificus* (proteobacterium, Jones and Oliver, 2009) or *Aureococcus anophagefferens* (pelagophyte, Zhang et al., 2012). The most straightforward quantitative monitoring technique available for the specific detection of viable picoplanktonic communities consists in whole-cell fluorescence *in situ* hybridization of rRNA (FISH, see Amann and Fuchs, 2008). This whole cell molecular assay allows also a precise localization of specific microorganisms within a biotic or abiotic substrate, this visualization is often necessary to study species interactions or micro-ecosystem functioning (e.g., Alverca et al., 2002; Biegala et al., 2005). Both absolute quantification and precise localization are advantages offered by whole cell FISH assays which are complementary to many valuable “omics” approaches which have high throughput in phylogenetic and metabolic diversity, but require cellular destruction (Rastogi and Sani, 2011).

FISH molecular assay uses fluorescently labeled oligonucleotide probes designed against more or less conserved zones of the rRNA sequence, which allows tagging populations at different taxonomic levels, from the domain to the strain (Groben and Medlin, 2005). Strong FISH signals are obtained from high ribosome content (up to 72,000 cell^-1^ in *Escherichia coli*, Bremer and Dennis, 1996). RNA content might, however, be lower in very small and slow-growing species, or in suboptimal growth conditions found in natural seawater, compared to marine bacterial cultures (Lee and Kemp, 1994; Kerhof and Kemp, 1999). The sensitivity of cellular RNA detection may therefore be increased by Tyramide Signal Amplification (TSA-FISH, Schönhuber et al., 1997), also called CAtalyzed Reporter Deposition (CARD-FISH, Pernthaler et al., 2002). In this setting, the specific oligonucleotide is linked to a horseradish peroxidase (HRP) enzyme catalyzing the permanent deposition of many fluorescent tyramides in the probe surroundings. It allows detecting cells with as low as 8.7 target rRNA molecules per cell (Hoshino et al., 2008), as well as highly autofluorescent micro-organisms (Biegala et al., 2002). For all these reasons, TSA amplification is highly recommended for the microscopic detection of marine picoplankton.

In addition to detecting a large range of environmental microorganisms, the TSA-FISH assay is highly specific, provided that the design of the probe is quality-checked and the stringency of hybridization conditions is optimized. For a probe to be specific, it should match with all known sequences affiliated to the group of interest, while having at least one central mismatch with sequences of non-target organisms. “Central” mismatches are indeed known to destabilize the probe-rRNA complex, while “terminal” mismatches are less destabilizing, as they are located one to two bases away from the 5′ or 3′ ends of the probe sequence. The importance of published probe *in silico* specificity control on a regular basis is now well recognized, in the context of fast-growing rRNA sequence databases (Amann and Fuchs, 2008). To date, the curated SILVA rRNA databases are the only ones covering the archaeal, bacterial and eukaryotic domains (Quast et al., 2013).

The hybridization conditions should also be optimized on target and non-target strain isolates, to make sure that the probe only binds target cell rRNA. This is done by adjusting the stringency of the hybridization and wash solutions to selectively detach the probe from non-target sequences (e.g., Daims et al., 1999). In some cases, unlabelled competitors (nucleotide sequence with no HRP) are used to mask the rRNA site by matching non-target sequences with central mismatches and avoiding unspecific HRP-probe binding. The stringency conditions should be adjusted after each modification of the hybridization protocol, in particular following the probe adaptation from monolabeled-FISH to TSA-FISH (Amann and Fuchs, 2008). Significant differences are indeed observed between the melting curves of monolabelled-probes and HRP-probes with their target rRNA, using formamide concentration increments (Hoshino et al., 2008). However, most often are specificity optimizations barely published and environmental studies use FISH probes without re-evaluating their specificity (e.g., Manti et al., 2012; Thiele et al., 2012).

The specificity of some probes targeting the bacterial and eukaryotic domains classes or clades frequently found in the marine environment can be investigated in more details. The members of the *Eubacteria* domain were first enumerated in different oceanic water masses using the Eub338 probe (Amann et al., 1990; Herndl et al., 2005). This domain specific probe was shown to detect, on natural samples and in TSA-FISH conditions, marine alpha-, gamma-*Proteobacteria*, *Bacteroidetes*, and *Cyanobacteria* as well as plastids from 84% of picoeucaryotes (e.g., Biegala et al., 2002, 2005). Many planctomycetales and verrucomicrobiales species, having two and three mismatches with Eub338, were later targeted by the EubII and EubIII probes (Daims et al., 1999). In 2008, the three probes mix matched with 94% of the bacterial sequences available (Amann and Fuchs, 2008), but the mix was applied to TSA-FISH on natural samples without re-evaluating its specificity (Wilhartitz et al., 2007). When looking at a more detailed level of the taxonomic classification, *Alpha* and *Gammaroteobacteria* frequently dominate marine planktonic *Eubacteria* worldwide (e.g., Pommier et al., 2007). Marine *Gammaroteobacteria* are detected by the Gam42a probe, which has been validated in combination with the unlabelled Bet42a competitor (e.g., Manti et al., 2012; Thiele et al., 2012). Although the latter masks rRNA sequences with one central mismatch (Manz et al., 1992) it also leads to many false-positive and negative hits (Barr et al., 2010). So, do all the probes designed against the general group of Alpha*proteobacteria*, which were found to be either unspecific, incomplete or both (Manz et al., 1992; Amann and Fuchs, 2008). However, probes directed against *Alphaproteobacteria* subgroups are more specific, such as Roseo536R, targeting *Roseobacter*, the second most abundant marine clade after the worldwide distributed SAR11 clade (Morris et al., 2002). In contrast, the Roseo536R probe has been found to match 94% of the *Roseobacter* clade sequences (Brinkmeyer et al., 2000; Tolli et al., 2006), while no *in silico* specificity detail is available for the alternative Ros537 probe (Eilers et al., 2001), which is frequently used for CARD-FISH analysis of marine water samples (e.g., Pernthaler et al., 2002; Thiele et al., 2012). Ros537 was redundantly designed at the same time as Roseo536R, and their sequences overlap, except that Ros537 is one nucleotide shorter. The need for *Roseobacter* probe specificity controls has become important since the clade diversity has increased 3.6 times during the last decade (Buchan et al., 2005, Silva SSU Ref #114).

When considering eukaryotic probes, the Euk1209 domain probe is frequently used (Giovanonni et al., 1988), although its specificity has never been controlled in TSA-FISH conditions, and it misses a significant part of the picoeukaryotic diversity (Not et al., 2002). The use of the Chlo01+NChlo01 mix (Simon et al., 1995; Not et al., 2002), later complemented with Euk1209 (Not et al., 2004), proved most efficient in quantifying marine picoeukaryotes. Among them, pelagophytes (*Stramenopiles*) and chlorophytes (*Archaeplastida*), are to date the most diverse and abundant taxa in oligotrophic oceans worldwide (Vaulot et al., 2008; Worden and Not, 2008). The Pela01 and Chlo02 probes designed for their detection have been used for TSA-FISH natural community quantification (Not et al., 2002; Biegala et al., 2003), but their specificity has not been controlled in a decade. Nor has that of Pras04 (Not et al., 2004), targeting the recently revised *Mamiellophyceae* class, a ubiquitous subgroup of picoplanktonic chlorophytes (Vaulot et al., 2008).

The objective of the present study was to re-evaluate a selection of probes and mixes, for the precise detection of the largest marine *Eubacteria* (Eub338, EubII, and EubIII) and picoeukaryote domains (Euk1209, NChlo01, Chlo01 and Chlo02), and of restricted groups of interest within these domains (Gam42a and competitor, Roseo536 and competitor, Roseo 537, Pela01, Pras04). This was done by (i) checking *in silico* that probes still target their group of interest, (ii) looking for the presence in recent databases of sequences from the closest outgroup (non-targeted) rRNA sequences, against which the probes need to be validated to be specific, (iii) adjusting the hybridization conditions on cultured strains, to bind only targeted cells, (iv) verify, when necessary, the new conditions on samples from the natural environment. We finally come up with a step by step comprehensive protocol for precise evaluation of probes specificity.

## Materials and Methods

### Specificity Evaluation *In Silico*

Each probe was tested under http://www.arb-silva.de/search/testprobe/against the curated SILVA “SSU Ref” or “LSU Parc” databases, for deposited sequences having up to three mismatches (Quast et al., 2013). The #114 database was released 2 months after #113 web release (Supplementary Table S1). The results of the ARB-SILVA “testprobe” analyses were filtered under Microsoft Office Excel^®^, deleting double entries (repeated accession number indicating different matching sites with the same number of mismatches). To look for potential unwanted matches with other rRNA types, probes directed against the SSU were tested against the “LSU parc” database, and LSU probes against the “SSU ref” database. Potential unwanted matches with known mRNA sequences were searched in the NCBI “*nucleotide collection nr/nt*” (GenBank + EMBL + DDBJ + PDB + RefSeq) database using the BLASTN 2.2.27+program (Johnson et al., 2008)^1^. Genomic sequences were not counted as matches, nor were mRNA sequences of organisms unexpected in marine plankton sample (e.g., mRNA sequence of the hen *Gallus gallus*). The matching list was therefore screened for sequences belonging to cultured strains, to test the probe specificity (**Table 1**, “Control strain”: (–)pCtrl).

**Table 1:** List of the probes used in this study, with the stringency parameters for the hybridization (formamide %) and washing (NaCl concentration) steps.

Probes	Specificity	Reference	Probe sequence Matching rRNA sequence Reversed control strain sequence	Specificity Control		Formamide [%] Tested	NaCl [mmol L^-1^] Tested
				(–)pCtrl rDNA	Accession		
Eub338	Domain	Amann et al., 1990	**5′ GCT GCC TCC CGT AGG AGT 3′** 3′ CGA CGG AGG GCA UCC UCA 5′ 3′ --- --- T-- --- --- A-- 5′	*V. spinosum*	X90515	50,55,**60,65**	18,10,**4,0**
EubII	Domain	Daims et al., 1999	**5′ GCA GCC ACC CGT AGG TGT 3′** 3′ CGU CGG UGG GCA UCC ACA 5′ 3′ --A --- A-- --- --- T-- 5′	*R. denitrificans*	M59063	**50,65**	**18,0**
EubIII	Domain	Daims et al., 1999	**5′ GCT GCC ACC CGT AGG TGT 3′** 3′ CGA CGG UGG GCA UCC ACA 5′ 3′ --- --- A-- --- --- T-- 5′	*R. denitrificans*	M59063	50,55,60,**65**	18,10,4,**0**
Gam42a	Class	Manz et al., 1992	**5′ GCC TTC CCA CAT CGT TT 3′** 3′ TT TGC TAC ACC CTT CCG 5′ 5′ -- --- -A- --- --- --- 3′	*H. muralis*	FN257757	**50**,55	**18**,10
Roseo536R	Clade	Brinkmeyer et al., 2000	**5′ CAA CGC TAA CCC CCT CCG 3′** 3′ GCC TCC CCC AAT CGC AAC 5′ 5′ --- --- --- C-- --- --- 3′	*P. aestuarii*	EF660757	50,55,**60**	18,10,**4**
RoseoC536R	Competitor	Brinkmeyer et al., 2000	5′ CAA CGC TAG CCC CCT CCG 3′			
Euk1209	Domain	Giovanonni et al., 1988	**5′ GGG CAT CAC AGA CCT G 3′** 3′ G TCC AGA CAC TAC GGG 5′ 5′ T --- --A --T --- --- 3′	*Haloarcheon* msnc14(3)	FJ868734	**40**	**46**
NChlo01	Division	Simon et al., 1995	**5′ GCT CCA CTC CTG GTG GTG 3′** 3′ GTG GTG GTC CTC ACC TCG 5′ 5′ --- --- --- -C- --- --- 3′	*Micromonas sp.*	DQ025753	**40**	**46**
Chlo02	Division	Simon et al., 2000	**5′ CTT CGA GCC CCC AAC TTT 3′** 3′ TTT CAA CCC CCG AGC TTC 5′ 5′ --- --- --- --A --- --- 3′	*L. reticulosa*	EF622539	**40**	**46**
Pela01	Class	Simon et al., 2000	**5′ ACG TCC TTG TTC GAC GCT 3′** 3′ TCG CAG CTT GTT CCT GCA 5′ 5′ --- A-- T-C --- --- T-- 3′	*S. scintillans*	Support. Info.	**40**	**46**
Pras04	Class	Not et al., 2004	**5′ CGT AAG CCC GCT TTG AAC 3′** 3′ CAA GTT TCG CCC GAA TGC 5′ 5′ --- --- --- A-- --- --- 3′	*P. subviridis*	U14386	**40**	**46**

*(–)pCtrl, negative probe control strains with mismatches. Bold means validated formamide concentrations.*

### Cell Cultures and Sample Preparation

Analytical and cell-culture grade biochemicals, Sartorius and Whatman Poly-Carbonate Track-Etched filtration membranes were purchased from Sigma–Aldrich (PCTE, Saint-Quentin Fallavier, France) and Dominique Dutscher (Brumat, France). Culture media for *Eukarya* were from the National Center for Marine Algae and Microbiota (NCMA, East Boothbay, Maine, United States) or the Roscoff Culture Collection (RCC, Roscoff, France). The natural seawater picoplanktonic sample (0.2–3.0 μm size-fraction) was collected on a 0.2 μm-pore 47mm PCTE filter after pre-filtration on 3.0 μm- and 10.0 μm-pore PCTE filters of 250 mL of water collected at the deep chlorophyll maximum 100 m deep from the open ocean South Pacific Ocean Time-series (SPOT) observatory near New Caledonia (october 2014). Triplicates 1/16th filter portions were hybridized with bacterial probes under different conditions described below. HRP-labeled oligonucleotide probes were from Thermo Fischer Scientific GmbH (Ulm, Germany), the TSA Plus Fluorescein Evaluation Kit from Perkin Elmer SAS (Courtaboeuf, France) and the Citifluor AF1 from Biovalley (Montpellier, France).

Cultured strains (Supplementary Table S2) were selected to belong either to the group targeted by the probe (positive probe control abbreviated as (+)pCtrl) or to an outgroup with the closest rRNA sequence having one to three mismatches with the probe (negative probe control abbreviated as (–)pCtrl, **Table 1**). (–)pCtrl strains were available within culture collections for all the tested probes except for Pela01, for which the closest outgroup strain available had four mismatches. Information on control strains used in this study is summarized in Supplementary Table S2. *Haloarcheon* msnc14(3) was grown on a liquid medium, while bacterial media were supplemented with 15 g L^-1^ agar, and prepared in distilled water according to DMSZ instructions (Supporting Information). Cells were harvested in early stationary growth phase. Cultures were fixed for 15 min at room temperature with 1% buffered paraformaldehyde (PFA, w:vol) final concentration, and clumps of cells were disaggregated by vortexing when needed, prior to immobilization on 0.2 μm-pores 47 mm PCTE membranes, embedding in low-gelling point 0.4% agarose and storing in absolute ethanol at –80°C (detailed in Supporting Information).

### Probe Specificity Adjustment on Cell Cultures

Probe specificity on control strains was tested first using the formamide concentrations recently validated [if possible in TSA-FISH conditions according to Biegala et al. (2002) and Biegala and Raimbault (2008), **Table 1** and **Figure 3**]. If the (–)pCtrl displayed a positive signal, formamide concentration (i.e., stringency) was increased by 5% increments until past the melting curve inflexion point, which gave a negative signal with the (–)pCtrl, and a positive signal with the (+)pCtrl (**Table 1**). Hybridization steps are briefly mentioned below, when detailed procedure is provided in Supplementary Materials. Cells were fixed, dehydrated and perforated, when needed, for the HRP-probe to pass through the cell wall, and hybridized with a HRP-coupled oligonucleotidic probe (**Table 1**). Probe hybridization was revealed by a TSA reaction using fluorescein-labeled tyramide (FITC, green fluorescence) and cellular DNA was DAPI-stained (blue fluorescence). The stringency of the hybridization conditions was optimized by adjusting the concentrations of formamide in the hybridization buffer (and of salt when necessary) in the washing buffer. Increasing stringency contributed to detach the oligonucleotide from the (–)pCtrl. We started with concentrations of 40 and 50% formamide for eukaryotic and prokaryotic probes, respectively.

### Microscopy

**FIGURE 1:**
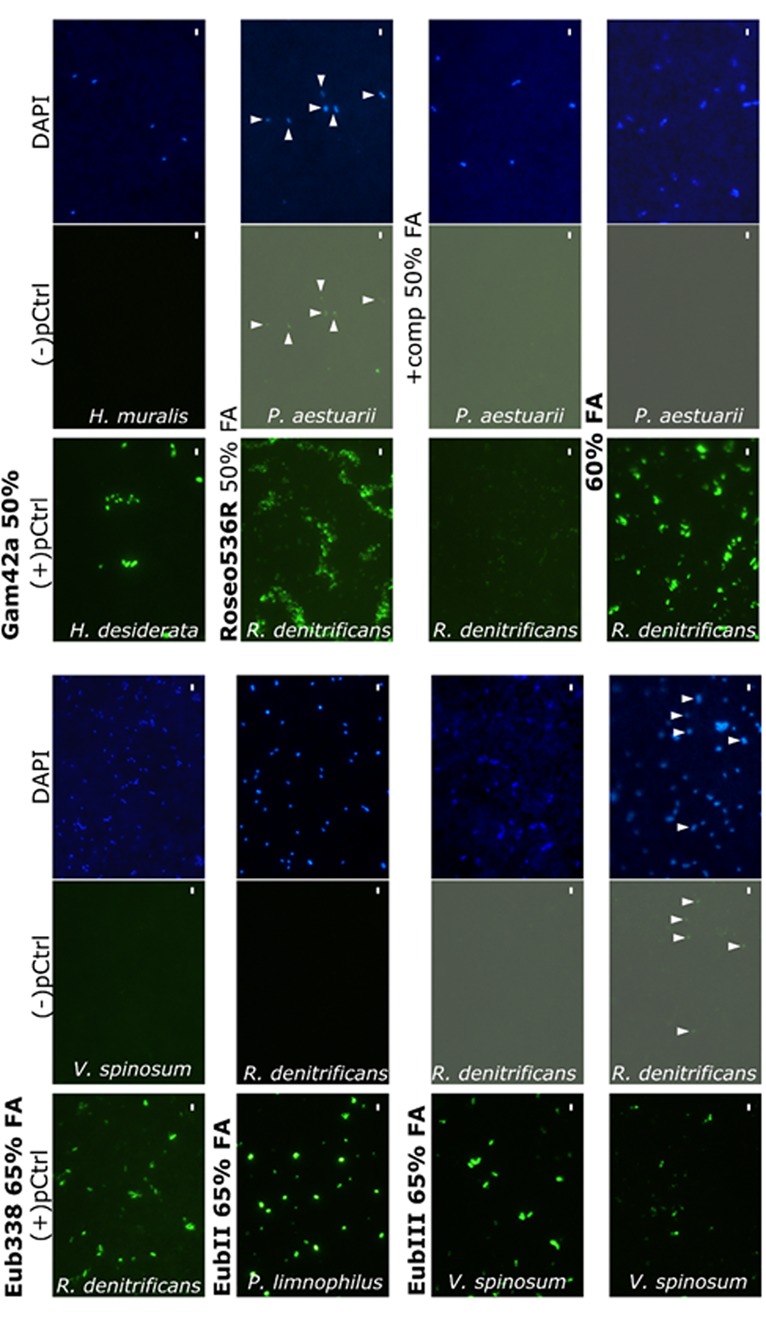
**Validation of the probes for the detection of marine bacteria.** (+)pCtrl are displayed in bold. (–)pCtrl unspecific signals are in normal text. The pictures presenting gray backgrounds have been modified with luminosity and contrast increases of 50 and 40%, respectively, and the arrows point at unspecific labeled cells. Scale bars indicate 2 μm.

Images were acquired at 100× magnification (40× for *L. reticulosa*) with an epifluorescence ECLIPSE 50i microscope (Nikon) equipped with excitation and emission dichroïc filters for DAPI and FITC detection, and a digital camera (see details in Supplementary Materials, QICAM 12-bit color cooled, QImaging). Pictures time exposure was an essential parameter to be defined in order to conclude on the success of a hybridization experiment. Time exposure was thus set up in order to reach saturation (i.e., the best picture, **Figures 1**, **3**) on the (+)pCtrl and then kept constant for the (–)pCtrl and other strain controls abbreviated as (+)sCtrl and (–)sCtrl (Supplementary Figure S1). However, we permitted a decrease in time exposure on the (+)sCtrl when the emission fluorescent signal was over-saturating preventing the presentation of results (Supplementary Figure S1), and some (–)pCtrl pictures were over-exposed to illustrate slight positive unspecific signals (**Figure 1**, arrows). FITC exposure times ranged from 600 ms for most probes to 1.2 s for Gam42a, 1.5 s for Pela01, and 2 s for Roseo536R. Camera colors were preserved, and no image processing was done on FITC pictures, except for the abovementioned over-exposed (–)pCtrl pictures (**Figure 1**), where brightness and contrast were increased as detailed in the figure caption, to allow proper visualization. In contrast DAPI exposure and image processing were adapted and processed to allow best picture to be presented (**Figure 1**). DAPI exposure ranged from 200 ms to 2.8 s, and DAPI pictures were processed where needed using Microsoft Office Picture Manager^®^ to optimize the brightness, contrast and Gamma parameters (**Figures 1**, **3**, and Supplementary Figure S1).

### Statistical Analyses

Differences in cell concentrations measured on natural seawater samples were analyzed by the non-parametric pairwise Mann–Whitney test on raw data (i.e., individual replicate values), due to the low number of replicates, using the Statistica 6 software. Data are reported as averages and standard deviation (SD) and statistical difference was accepted at *p* ≤ 0.05.

## Results

### Specificity of Eubacterial Probes

Among the six studied prokaryote probes and competitor, only EubII and EubIII remained fully specific, meaning they were 100% complementary to sequences affiliated to Eubacterial, targeted sequences (none sequences in outgroup column at zero mismatches, Supplementary Table S1). Eub338 alone targeted 93.3% of the SSU Ref #114 Silva database sequences affiliated to *Bacteria* with a perfect match or one terminal mismatch, missing 99.9% of the sequences classified as *Verrucomicrobiales* and *Planctomycetales* (Supplementary Figure S2). When EubII and EubIII target sequences (88% of *Verrucomicrobiales* and 49% of *Planctomycetales* affiliated sequences, respectively), were added to those of Eub338, the coverage of *Eubacteria* affiliated sequences increased up to 96%. The Eub338+EubII+EubIII mix was specific to the *Eubacteria* domain with 586338 hits, while only two outgroup hits affiliated to salt-water *Archaea* were targeted by Eub338. Other probe mix outgroup hits in the database harbored either one central mismatch with Eub338 and EubIII (“30c”, Supplementary Table S1), or two mismatches with EubII. Except for two of these outgroup sequences being affiliated to coastal air fungi, the other sequences were 18S rRNA sequences amplified from terrestrial samples (9 sequences), or affiliated to marine species harboring plastids with target 16S rRNA (chlorarachniophytes, 23%) or over 20 μm in size (70%). The majority of closest outgroup sequences had two to three mismatches with the probe mix: they were affiliated with *Archaea* (“28tc”, “211ttc”, Supplementary Table S1) and *Eukarya* (“745cc” including 46 sequences affiliated to uncultured marine *Ciliophora*).

**FIGURE 2:**
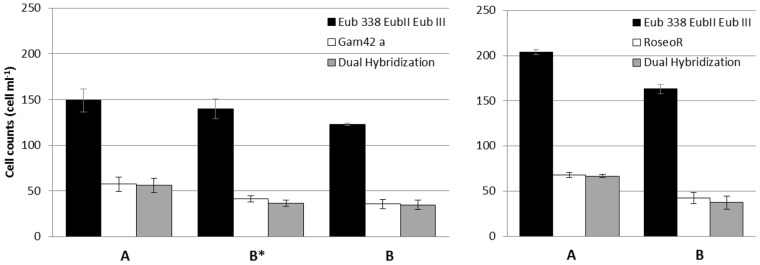
**Comparison of cell counts between hybridization conditions of prokaryotic probes (A)** recommended (this study), and **(B^∗^,B)** from the literature. **(B^∗^)** Show counts with Gam42a probe without the competitor. Counts on 10 optical fields from triplicates are averaged ±SD.

When tested on pure cultured strains, EubII was already specific to (+)pCtrl with zero mismatch (**Figure 1**) and one mismatch (*Verrucomicrobium spinosum* DSM 4136, data not shown) at 50% formamide, and Eub338 was specific to strains with (+)pCtrl at 60% formamide (**Table 1** and **Figure 1**). However, it was necessary to increase the concentration to 65% to obtain a complete negative signal with EubIII on the control cells displaying two to three mismatches. This result imposed the use of 65% formamide as specific conditions for the Eub338+EubII+EubIII mix). At 65% formamide, EubII detached from target sequences with one central mismatch (*Verrucomicrobium spinosum* DSM 4136, not shown). However, 99% of these sequences were targeted by EubIII. When tested on natural environment samples, these changes in formamide concentration (65%) induced significantly (Mann–Whitney *p* = 0.025, *N* = 15) higher counts than conditions previously described in the literature (55%, **Figure 2**).

The Gam42a probe matched with 84% of the 23S rRNA sequences affiliated to gamma-*Proteobacteria* in the LSU Parc #114 database (Supplementary Table S1). Out of the 6639 sequences matching perfectly with the probe 0.5% were outgroup hits, half of which being affiliated to alpha*-* and *beta-Proteobacteria*. Sequences with one central mismatch were at 12% classified as target *gamma-Proteobacteria*, but they also included many sequences affiliated to non-target *alpha-* and beta*-Proteobacteria*. The unlabelled Bet42a competitor matched with 91% of the outgroup hits with one central mismatch, but also prevented the detection of 8% of target gamma-proteobacteria harboring one central mismatch.

Concerning the alpha-proteobacteria *Roseobacter* clade, 91% of the identified member sequences were detected using both Ros537 and Roseo536R probes (Supplementary Table S1). These probes also targeted 2.9% of sequences classified as non-*Roseobacter Bacteria*, mostly (73%) other *Rhodobacterales alpha-Proteobacteria*, which may be found in marine samples. The RoseoC536R competitor masked sequences with one central mismatch, including 99.7% of the sequences identified as outgroup hits, but also 6% of the identified *Roseobacter* clade sequences. For both group-specific probes Gam42a and Roseo536R, specificity was reached without competitors (**Figure 1**). The original stringency of 50% formamide was confirmed for Gam42a, when it had to be increased up to 60% for Roseo536R (**Figure 1**). When tested in the natural environment significantly higher counts were obtained with Roseo536R used at 60% formamide compared to the former use of 50% formamide and competitors (Mann-Whitney *p* = 0.049, *N* = 6), and the addition of Bet42a competitor did not affect the proportion of cells detected by Gam42a (Mann–Whitney *p* = 0.154, *N* = 9, **Figure 2**). All the cells hybridized by the group-specific probes Gam42a and Roseo536R were also detected by the mix Eub338+EubII+EubIII in the conditions validated in the present study.

### Specificity of Probes Targeting Pico-Eukaryotes

**FIGURE 3:**
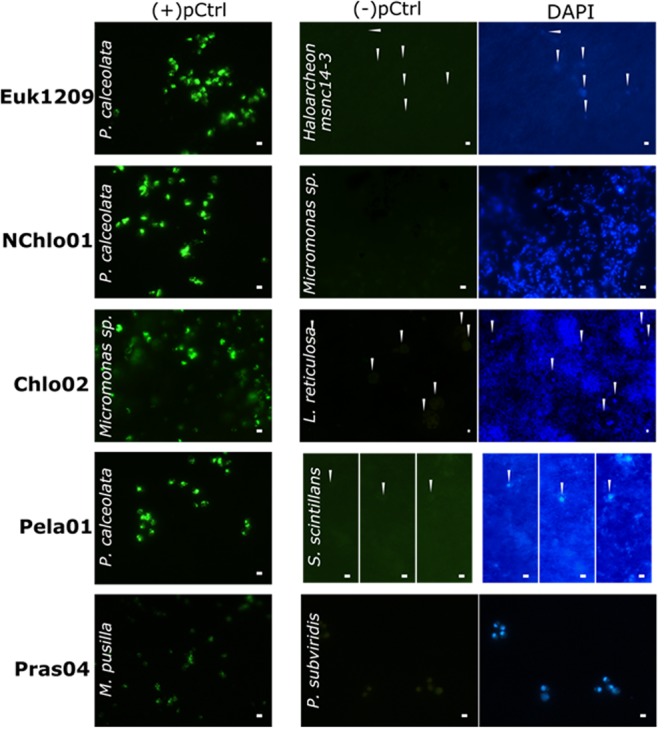
**Validation of the probes for the detection of marine pico-eukaryotes.** (+)pCtrl include 0 mismatch strains labeled with FITC (green)(–)pCtrl are specificity controlled strains. Thin white arrows point at the location of unspecific labeling of (–)pCtrl control cells. DNA counterstaining is labeled by DAPI (blue). Scalebars indicate 2 μm.

Among the five eukaryotic probes and the six mixes analyzed, the Euk1209, Pela01 probes and the mixes Chlo02+NChlo01, Euk1209+Chlo02, Euk1209+NChlo01, Euk1209+NChlo01+Chlo02 were fully specific (Supplementary Table S1). However, the Euk1209 probe covered the eukaryotic domain only partially (Supplementary Figure S3) as it missed 4223 sequences classified as eukaryotes with one central mismatch, including 277 sequences of marine planktonic species (Supplementary Table S1). The two outgroup hits with two central mismatches were affiliated to uncultured *Bacteria* and *Archaea*. Euk1209 also had three central mismatches with many outgroup aquatic bacterial sequences and *Archaea* including the cultivated *Haloarchaeon* msnc14, used as the closest (–)pCtrl strain (**Figure 3**). The non-chlorophyte NChlo01 probe complemented the Euk1209 targets with 3432 hits, including 531 sequences affiliated to planktonic organisms. The NChlo01 probe was, however, not specific to the non-chlorophyte divisions, matching perfectly with 111 chlorophytes, and it had one central mismatch with 45 sequences of mostly marine uncultured organisms identified as *Archaea*. Chlo01 probe, complementary to NChlo01 matched with zero mismatches with three *Archaea* outgroup sequences. When used as a mix with Euk1209+NChlo01, Chlo01 decreased the specificity of the mix by adding three outgroup hits with zero mismatches (Supplementary Table S1). Chlo01 was also far from being specific to the *Chlorophyta* division when used on its own as it showed perfect match with 2173 and 211 sequences affiliated to non-chlorophyte *Alveolata* and *Heterokonta*, respectively. In contrast, Chlo02 matched with 81% of the sequences identified as *Chlorophyta* (Supplementary Table S1) and matched with only seven sequences linked to small planktonic non-chlorophyte eukaryotes. Yet, the hybridization of many outgroup sequences from planktonic organisms having one central mismatch with Chlo02 may be an additional source of false positives. However, when added to the Euk1209+NChlo01 mix, the Chlo02 probe added 109 target hits to the mix without adding any outgroup hits to the mix contrary to Chlo01 probe.

The pelagophyte class includes the *Pelagomonadales* and *Sarcinochrysidales*, with 100 and 92% of the sequences affiliated to these “orders”, respectively, matching with the Pela01 probe. Only two sequences of uncultured putative pelagophytes presented one and two central mismatches with the probe (Supplementary Table S1). Outgroup sequences, with two and three mismatches, were identified at 88% as non-target pluricellular rhodophytes and opisthokonts unlikely to be found in marine plankton samples, and at 11% as large marine *Radiolaria* (>100 μm). The remaining 1% were affiliated to uncultured picoplanktonic freshwater *Archaea*, *Amoebozoa*, or sporocyte-forming eukaryotes. Finally, Pras04 detected almost all (95%) the sequences classified as *Mamiellophyceae*, but also three non-target sequences affiliated to marine organisms (dinoflagellate, stramenopile and chrysophyte, Supplementary Table S1). It had one central mismatch with 17 target hits, and with many outgroups hits which may be found in marine planktonic samples (e.g., 416 sequences of stramenopiles, 219 sequences affiliated to non-*Mamiellophyceae* chlorophytes). At 40% formamide, all eukaryotic probes were specific to their (+)pCtrl, giving negative hybridization signals with (–)pCtrl having one to four central mismatches (**Figure 3** and **Table 1**). A slight brownish color was seen in the (–)pCtrl *P. subviridis* which show some autofluorescence of this photosynthetic strain.

## Discussion

### Specificity of Bacterial Probes

The Eub338+EubII+EubIII probe mix has been developed to extend the specificity of Eub338 to Planctomycetales and Verrucomicrobiales, which are abundant in fresh- and sea-waters. The mix has been used previously for the analysis of environmental samples in CARD-FISH at 35 or 55% formamide in the hybridization buffer (Wilhartitz et al., 2007; Thiele et al., 2012). In the present study, the positive Eub338 TSA-FISH signal obtained with the eubacterium *Verrucomicrobium spinosum* raises concerns about the specificity of these hybridization conditions. This strain indeed harbors a 16S rRNA sequence having two mismatches with Eub338, similarly as 28 archaeal sequences (“28 tc”, Supplementary Table S1). To reduce the risk for false positives it is necessary to increase stringency by increasing formamide concentration in hybridization buffer from 55 to 60% when using Eub338 probe at one mismatch specificity. Although some archaeal cell wall deprived of lysozyme target site, may not allow HRP-labeled probes to penetrate the cell and target 16S rRNA (Pernthaler et al., 2002), still, some *Euryarchaeota*, which belong to Archaeal kingdom, require no perforation for TSA-FISH assay (Schönhuber et al., 1997).

A further increase of formamide concentration up to 65% was found necessary to prevent the hybridization of EubIII with uncultured marine *Alveolata* (Ciliophores) harboring two mismatches with the probe. When tested on Eub338 and EubII probes specificity 65% of fomamide did not change positive signals on probe target from 60% formamide condition. Therefore, we recommend using the Eub338+EubII+EubIII probe mix at a formamide concentration of 65%. In the natural environment, such as the one used in this study (**Figure 2**), using 65% formamide resulted in higher cell counts than when 55% was used. This result was unexpected, since when stringency conditions are increased they are usually expected to reduce the amount of false positive species targeted by the probe, thus reducing the overall cell concentration.

Together with the *Gammaproteobacteria*, the *Alphaproteobacteria* can account for approximately 30 to 60% of the bacterial diversity in the world ocean (e.g., Pommier et al., 2007). Their respective proportions may result from environmental physico-chemical parameters, and influence local biogeochemistry (Falkowski et al., 2008). Since Gam42a is the only probe currently available to target the largest *Gammaproteobacteria* diversity, we validated the hybridization conditions to avoid its binding to sequences with one central mismatch. We found no use for the competitor Bet42a since Gam42a with or without its competitor show the same amount of target and outgroup at zero mismatches (Supplementary Table S1) and Gam42a on its own was specific at 50% formamide to its target and did not bind *H. muralis* with one central mismatches (**Figure 1**). Accordingly, with these results Gam42a alone or with its competitor targeted comparable cell numbers in a natural sample (**Figure 2**). However, the 55% formamide concentration used by Manti et al. (2012) was found to be too stringent, decreasing the positive signal intensity (data not shown). The relative specificity of Gam42a has not declined since its design (Manz et al., 1992; Amann and Fuchs, 2008; present study), but the classification of *Gammaproteobacteria* is experiencing some changes with the development of multi-gene phylogeny (Williams and Kelly, 2013). We found Gam42a to match for instance with 19 sequences of 23S rRNA affiliated to *Acidithiobacillales*, which were very recently excluded from the class *Gammaproteobacteria* (Williams and Kelly, 2013). This miss-identification may be problematic in marine environmental studies, since the *Acidithiobacillales* include strains isolated from seawater (Kamimura et al., 2003). This might call for the design of a new 16S rRNA probe in future studies.

Among *Alphaproteobacteria*, the *Roseobacter* clade is ubiquitously distributed from coastal to open ocean and from the surface down to the seafloor (Buchan et al., 2005). These members of the *Rhodobacteraceae* family were found to be key-players in the global carbon and sulfur cycles (Wagner-Döbler and Biebl, 2006). However, they are not easily brought to culture, stressing the need for specific culture-independent methods to evaluate their importance (Eilers et al., 2001). Within five years the 16S rRNA sequences from *Roseobacter* clade (Brinkmeyer et al., 2000), increased by one order of magnitude from 124 to 1497 sequences (Buchan et al., 2005), and reached 5440 (SSU Ref #114), calling for a re-evaluation of the probes *in silico* specificity. In addition, many “unidentified” sequences affiliated to uncultured *Rhodobacteraceae* have been discovered, which potentially belong to the *Roseobacter* clade. The specific stringency for Roseo536R defined by our study (60% formamide, without competitor) cannot avoid the hybridization of 1–3.4% of false positives. Using Roseo536R at 60% formamide prevents binding to many non-*Roseobacter Alphaproteobacteria* with one central mismatch. It also allows to have a much higher fluorescence on it target than when using former conditions (50% formamide + competitor). This observation may directly explain the significantly higher cell counts obtained in the natural environment with the new conditions than with former ones (**Figure 2**). This result might indicate that at 50% formamide, unspecific binding of the competitor on Roseo536R targets could prevent binding of the probe, and we therefore suggest that increasing the stringency is a better solution than using the competitor. Unfortunately, we found that it would miss 16% one mismatch sequences of uncultured *Roseobacter* members. Thus, similarly as Gam42a the design of FISH probes should be refined in further studies by targeting more specific groups.

### Current Specificity of Picoeukaryotic Probes

The specificity of Euk1209 had not been re-evaluated after its adaptation to TSA-FISH to detect dinoflagellates (Biegala et al., 2002), haptophytes and prasinophytes (Not et al., 2002; Biegala et al., 2003). The negative signal we obtained with an outgroup archaeal strain provides the first evidence that the former conditions were specific and may avoid the false positive detection of outgroup *Archaea* and *Bacteria* with two central mismatches or more. The latter should, however, not be a problem, since they can easily be distinguished from most eukaryotic cells showing a compact nucleus after DAPI staining, and since *Eukarya* do not require perforation for the penetration of an HRP-probe as do most *Archaea* and *Bacteria*. Unspecific TSA-FISH hybridization of prokaryotes should thus not happen when labeling eukaryotic cells with Euk1209. Nevertheless, since some *Archaea* do not need perforation as mentioned above (Schönhuber et al., 1997), and given that the marine archaeal diversity is still poorly known, it was important to make sure that Euk1209 was specific. This is important for example in the context of automatic detection, or of dual-hybridizations to detect eukaryotic intracellular *Bacteria* (Biegala et al., 2005). Previous studies stressed that Euk1209 needs to be complemented with other probes for a more exhaustive detection of the phytoplanktonic picoeukaryotic community (Not et al., 2004). We thus checked the current complementarity and specificity of the large spectrum probes NChlo01, Chlo01 and Chlo02 that had been designed against phytoplanktonic groups (Supplementary Table S1). We conclude that the Euk1209+NChlo01+Chlo01 mix (Not et al., 2004) should be replaced by the more specific Euk1209+NChlo01+Chlo02 mix, enlarging Euk1209 phytoplanktonic spectrum mostly with stramenopiles, alveolates, and chlorophytes. Recently, this mix has been used for the first time in a freshwater eukaryotic microbial community study (Mangot et al., 2013), but its specificity remained to be tested by *in silico* analyses and TSA-FISH with control cultures. Before our study, Chlo02 was known to be specific at 40% formamide to sequences with less than two mismatches (Biegala et al., 2003) which was sufficient for its use in the mix, but NChlo01 had only been validated in the presence of unlabelled Chlo01 differing by one mismatch (Biegala et al., 2003). Here we show that the same TSA-FISH stringency conditions avoid the hybridization of undesired outgroup sequences for each probe of the new mix.

The specific detection of the chlorophyte division by Chlo02 is hindered by outgroup hits consisting of matching sequences of <100 μm freshwater planktonic zygnematales, which can, however, easily be distinguished by their unique shape, during filter examination. We made sure for the first time that the TSA-FISH stringency conditions defined for Chlo02 in earlier studies (**Figure 3** and Supplementary Table S1) selected against the hybridization of many sequences with one central mismatch affiliated to non-target *Rhizaria*, alveolates, heterokonts, and haptophytes. Narrowing down to the class level detection, we observed that the TSA-FISH conditions for the *Mamiellophyceae* chlorophyte subgroup-specific probe Pras04, had been previously optimized against strains with rRNA sequences having two mismatches (Not et al., 2004). Our study shows that the same conditions allowed selecting against non-target non-*Mamiellophyceae* chlorophytes and stramenopiles with one central mismatch detected by the *in silico* analysis, but prevented the hybridization of 17 target sequences. Finally, the specificity of the Pela01 *Pelagophyceae* class-directed probe, previously validated against strains with more than five mismatches (Not et al., 2002; Biegala et al., 2003), could not be tested against outgroup hits with three central mismatches since they consisted in uncultured organisms.

### Step by Step Protocol for the Validation of TSA-FISH Probes

**FIGURE 4:**
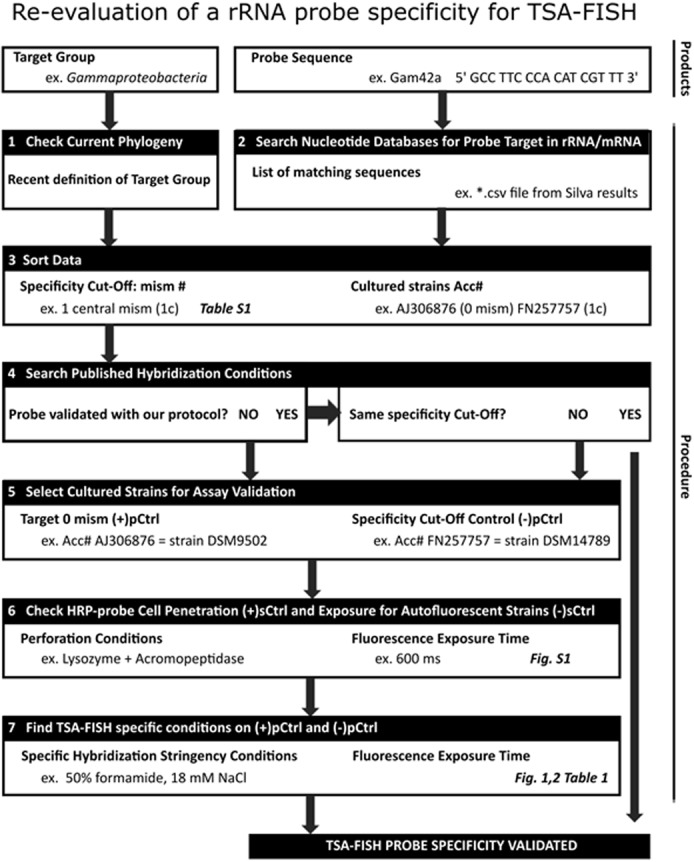
**Protocol steps for the re-evaluation of a probe specificity in TSA-FISH conditions before its application on environmental samples**.

We propose a schematic overview of the protocol (**Figure 4**) that was followed for each re-evaluation of general and group-specific probes in TSA-FISH assays. The specificity of published probes must be verified from time to time as nucleotide databases are growing exponentially which may comfort or refute their use in the natural environment:

STEP-1: The target phylogenetic group has to be well defined.
STEP-2: The list of probe-matching sequences resulting from database queries is then analyzed.
STEP-3: The outgroup hits at zero, one or two mismatches have to be thoroughly examined in case of their potential occurrence in targeted natural environment.
STEP-4: The specificity of each probe obtained by changing stringency conditions (i.e., percentage of formamide in hybridization buffer) has to be validated using the whole cell hybridization protocol planned to be used in the targeted natural environment.
STEP-5: If validation is needed, two control culture strains should be selected, a positive control, with a zero-mismatch target rRNA sequence and a negative control with target rRNA sequence including the amount of mismatches defined as specificity cut-off *in silico* analysis.
STEP-6: These control strains should be tested (i) for the penetration of the HRP-probe under membrane adapted perforation conditions, using a kingdom eukaryotic or prokaryotic probe (i.e., (+)sCtrl) and (ii) for their potential to generate autofluorescence (i.e., (–)sCtrl).
STEP-7: Only then can the probe validation be done, using adapted strains for (+)pCtrl and (–)pCtrl adjusting if needed formamide concentration in the hybridization buffer using fixed hybridization temperature and salt concentration in the washing buffer. As formamide concentration cannot be increased above 65%, higher stringency can be obtained by changing salt or temperature during hybridization. For micrograph proof of the cultures test, we advise that time exposure is defined on target or positive control culture and kept fixed on negative control culture as well as on positive control culture hybridized with kingdom probe used in STEP-6 (**Figures 1**, **3**, and Supplementary Figure S1).

The probe is then ready to be used on environmental samples.

## Conclusion

Defining the spectrum of rRNA probes and optimizing the experimental conditions to ensure their specific binding is important to improve the quality of population dynamics during environmental analyses. However, most environmental studies currently skip the probe specificity checking steps. During this investigation, we also observed that TSA-FISH assays are rarely optimized on cultured strains, and that the hazardous use of competitors is frequently adopted to prevent currently known non-targets to be labeled. We conclude that increasing the hybridization stringency should be preferred when possible to the use of unlabelled competitors, since false positives may arise from undesired environmental sequences, not yet reported in databases. We moreover stress the urgency to design more specific probes against alpha- and *gamma-Proteobacteria*. On the contrary, the refined hybridization specificity check done in this study confirmed that eukaryotic probes examined were all specific. This study shows that an in-depth specificity evaluation can be performed in a systematic manner (**Figure 4**) and received an ISO-9001 quality certification (version 2015). From time to time these four to seven steps procedure are advised to be included in the method section of research articles for commonly used probes, when it should be a prerequisite for the validation of new probes.

## Author Contributions

VR performed the *in silico* analysis and with MP acquired the culture samples and contributed to laboratory analysis. VR contributed significantly to all sections of the manuscript. MP acquired the samples from the natural environment. IB designed the study, organized the analysis, and contributed to the different sections of the manuscript.

## Conflict of Interest Statement

The authors declare that the research was conducted in the absence of any commercial or financial relationships that could be construed as a potential conflict of interest.

## Footnotes

1 http://www.ncbi.nlm.nih.gov/BLAST/Blast.cgi?PROGRAM=blastn&BLAST_PROGRAMS=megaBlast&PAGE_TYPE=BlastSearch&SHOW_DEFAULTS=on&LINK_LOC=blasthome
