# Supplementary material for: Specificity Re-evaluation of Oligonucleotide Probes for the Detection of Marine Picoplankton by Tyramide Signal Amplification-Fluorescent In Situ Hybridization
Source: Front Microbiol. 2017 May 29;8:854. doi: 10.3389/fmicb.2017.00854 (PMC5446981; doi:10.3389/fmicb.2017.00854)
Supplement: Supplementary file 1 [file Data_Sheet_1.pdf]

**Supplementary Material**

# Specificity re-evaluation of oligonucleotidic probes for the detection of marine picoplankton by Tyramide Signal Amplification-Fluorescent *in situ* Hybridization

Riou Virginie[1], Périot Marine[1], Biegala Isabelle C.[1,*]

[1]Aix Marseille Université, Université de Toulon, CNRS, IRD, Mediterranean Institute of Oceanography (MIO), Marseille, France.

*isabelle.biegala@ird.fr

**Supporting data:** *Symbiomonas scintillans* RCC257 18S ribosomal RNA gene, source = RCC. Highlighted zones indicate the binding sites for Pela01 (dark blue), Chlo02 (light blue) and NChlo01 (grey), with mismatches or deletions highlighted in red.

**5'**CTCGTAGTTGAACTTCTGAGCCACGTGCATGGGCGCCACACATGTGGCCCCTCA
CTGTGGCTCATCTTCGTTGTTGAACCTTGGCGGCGCAAGTCACCTTGGGGATCGG
CGTCATTTACTGTGAAAAAATTAGAGTGTTCAAGGCAGGCGTTAGCCCGTATCCA
CTAGCATGGAATAATAAGATAGGACCTCGTGCTATTTTGTTGGTTGCGGTTCGAG
GTAATGATTAATAGGGACAGTTGGGGGTATTCGTATTTAGCTGTCAGAGGTGAAA
TTCTTAGATTTGTTAAAGACGAACGACTGCGAAAGCATCTACCAAGGATGTTTTC
ATTAATCAAGAACGAAAGTCAGGGGATCGAAGAGGATTAGATACCCTCGTAGTC
CTGTACCATAAACGATACCGACCCGCGATTGTCAGGTGTATTCAAGACCCTGGCA
GCAGCGAAGAAGAAATTCCGAGTCTTTGGGTTCCGGGGGGAGTATGGTTGCAAA
GCTGAAACTTAAAGGAATTGACGGAAGGGCACC*CAAGAAGTGGAGCCTGCGGC
TTAATTCGACTCAACACGAGAAAACTTACCAGGTCCGGACAAAACAAGGATTGA
CAGTTTGAGAGTACTTTCTTGATTTTTTGGTTGGTGGTGCATGGCCGTTCTTAGTT
GGTGGAGTGATTTGTCTGGTTGATTCCGATAACGAACGAGACCTTCGCCTGCTAA
TTAGTTCTCTCCCCTACCGGGGTATTGAGGACTTCTTAGAGGGACTCGTT**-3'**

## Cell cultures

*Haloarcheon* msnc14(3) was grown on the medium defined by Tapilatu *et al.* (2010), in which the agar was omitted. All bacterial media were supplemented with 15 g $L^{-1}$ agar and prepared in distilled water according to the DMSZ instructions: medium 545 was prepared from commercial Tryptic Soy Broth (Sigma #22092), medium 514 from bacto marine broth (DIFCO 2216), while media 621 (PYGV AGAR), 607 (M13 *Verrucomicrobium* Medium), 762 (*Halomonas desiderata* Medium: Glucose 5.0 g $L^{-1}$ $MgCl_2$ x 6 $H_2O$ 0.2 g $L^{-1}$, $KH_2PO_4$ 1.0 g $L^{-1}$, $KNO_3$ 2.0 g $L^{-1}$, $Na_2CO_3$ 5.4 g $L^{-1}$, $NaHCO_3$ 4.2 g $L^{-1}$, final pH 9.5-10.0), were prepared by assembling the different chemicals. The eukaryotic L1-Si and K media were prepared from commercial kits, and the « Riz » (Rice) medium was provided by the RCC.

**Sample preparation**

The 10% buffered paraformaldehyde (PFA, w:vol) stock solution was prepared by adding 10 g of PFA to 65 mL warm (56°C) sterile MilliQ water under stirring. Dissolution was obtained by warming and adding few drops of concentrated NaOH (10N) to obtain a translucent solution. The solution was then removed from the heating plate, 10 mL of 10% Phosphate Buffered Saline pH 7.4 was added, and pH was adjusted to 7.2, before adjusting the volume to 100 mL with sterile MilliQ water and filtering the preparation on chemistry paper filter. Aliquots of 10 mL stock 10% PFA were stored at -20°C up to 1 year or at 4°C for up to 3 weeks to avoid formaldehyde dismutation.

Strain RCC375 and all bacterial strains were vortexed after fixation to disaggregate clumps of cells. After this step, the fixed cells may be quickly frozen at -80°C until further processing. Freshly fixed cells or frozen aliquots (limited to one freeze-thaw cycle) were then collected onto 0.2 µm-pores 47 mm PCTE filters at a concentration allowing the detection of 10-50 cells per camera field (depending on the size of the cells). The cells were further embedded on the filter in low-gelling point 0.4% agarose ([wt/vol] in Milli-Q water), dried at 37°C for 15 min, dehydrated for 10 min in 100% ethanol and quickly frozen at -80°C until further analysis.

**Cell perforation and hybridization by TSA-FISH**

Picoeukaryotes did not need any perforation treatment for the penetration of the HRP. Bacterial cell walls were perforated as in Biegala & Raimbault (2008), with slight modifications, using a 60 min treatment at 37°C with ≈500,000 U $ml^{-1}$ lysozyme (10 mg $mL^{-1}$, ref. L6876 ≈40,000 U $mg^{-1}$) in 0.05 M $Na_2EDTA$ [pH 8.0], 0.1 M Tris-HCl [pH 7.5]. It was followed by three rinsing steps of 5 min in 5 mL Milli-Q sterile water. Then, a 30 min incubation at 35°C with 60 U $mL^{-1}$ achromopeptidase (ref. A3547 ≈4,500 U $mg^{-1}$, Sekar *et al.*, 2003) in 0.01 M NaCl, 0.1 M Tris-HCl [pH 7.5], was followed by three rinsing steps of 5 min in 5 mL Milli-Q sterile water.

The perforation of archaeal cell walls was done as in Teira *et al.* (2004) for 60 min at 37°C with 4 U $ml^{-1}$ proteinase K (6.7 µL $mL^{-1}$ of a 600 U $mL^{-1}$ solution, ref. P4850) in 0.05 M $Na_2EDTA$, 0.1 M Tris-HCl [pH 8.0] (Teira *et al.*, 2004). After this treatment, filters were rinsed three times in 5 mL sterile Milli-Q water, incubated for 20 min at room temperature in 5 mL 0.01 M HCl and washed twice with 5 mL Milli-Q water.

Before the hybridization step, 1:16 filter portions were soaked for 10 min in 100% ethanol and dried 15 min at 37°C. The hybridizations with 5'-horseradish peroxidase (HRP)-labeled oligonucleotide probes (5 ng DNA µ$L^{-1}$, final oligonucleotide concentration) using the formamide concentrations detailed in Table 2, were performed for 2h at 35°C as in Biegala *et al.* (2002), except for *Haloarcheon* msnc14(3), which was incubated overnight with Arch915 (Stahl and Amman, 1991, 5% formamide, Ushio *et al.*, 2013), with Euk1209 or without probe as a "no probe control". After the hybridization, filters were washed twice for 30 min at 37°C in 5 mL of prewarmed washing buffer consisting of 0.02 M Tris-HCl [pH 7.5], 0.01% Sodium Dodecyl Sulfate, 5 mM $Na_2EDTA$ [pH 8] and NaCl concentrations listed in Table S2 (according to Amann and Schleifer, 2005). Equilibration in Tween-NaCl-Tris buffer (TNT), TSA reaction and TNT rinsing steps were done as in Biegala *et al.* (2002), using the TSA Plus Fluorescein Evaluation Kit (green fluorescent emission). Filters were then allowed to

equilibrate for 10 min at room temperature in sterile 1X PBS [pH 7.4], their down-side was wiped dry before DNA staining as in Biegala *et al.* (2002) on eubacteria and eukaryotes, for 15 min in the dark by 10 µL of a 5 µg $mL^{-1}$ DAPI (4',6-diamidino-2-phenylindole dihydrochloride, blue fluorescent emission) mixture consisting in two volumes of 10 µg $mL^{-1}$ aqueous DAPI solution, one volume of 1X PBS and one volume of AF1 (Citifluor Ltd.). After a 10 min washing step in sterile 1X PBS [pH 7.4], filters down-side were wiped dry, and they were mounted in AF1 anti-fading reagent (Citifluor Ltd.), and stored at 4°C until further microscopic analysis.

**Microscopy**

Images were acquired with an epifluorescence *ECLIPSE 50i* microscope (Nikon) equipped with an Halogen lamp (6V 30W, H65761, Orbitec), two objectives (40X: NA 0.75N Plan Fluor WD 0.72mm, Nikon; and 100X: NA 1.30N Plan Fluor WD 0.16mm, Nikon), excitation (ex.) and emission (em.) dichroïc filters (365±10 nm ex., 400 nm em. for DAPI detection; 480±40 nm ex., 510 nm long pass em. for the Fluorescein IsoThioCyanate FITC detection) and a digital camera (QICAM 12-bit color cooled, QImaging). All the pictures were taken at 100X magnification, except for *L. reticulosa* (40X).

**References**

Amann, R.I., and Schleifer, K.H. (2005) Nucleic Acid Probes and Their Application in Environmental Microbiology. *In* Bergey's Manual® of Systematic Bacteriology, Volume One, The *Archaea* and the Deeply Branching and Phototrophic *Bacteria.* Boone, D.R., and Castenholz R.W. (eds). Springer-Verlag New York.

Biegala, I.C., Kennaway, G., Alverca, E., Lennon, J., Vaulot, D., and Simon, N. (2002) Identification of bacteria associated with dinoflagellates (Dinophyceae) *Alexandrium spp.* using tyramide signal amplification-fluorescent *in situ* hybridization and confocal microscopy. J. Phycol., 38, 404-411.

Sekar, R., Pernthaler, A., Pernthaler, J., Warnecke, F., Posch, T., and Amann, R. (2003) An improved protocol for quantification of freshwater Actinobacteria by fluorescence in situ hybridization. Appl. Environ. Microbiol., 69, 2928-35.

Stahl, D.A., and Amann, R. (1991) Development and application of nucleic acid probes. In E. Stackebrandt and M. Goodfellow (ed.), Nucleic acid techniques in bacterial systematics. John Wiley & Sons Ltd., Chichester, England, 205-248.

Tapilatu, Y.H., Grossi, V., Acquaviva, M., Militon, C., Bertrand, J.C., and Cuny, P. (2010) Isolation of hydrocarbon-degrading extremely halophilic archaea from an uncontaminated hypersaline pond (Camargue, France). Extremophiles, 14, 225-231.

Teira, E., Reinthaler, T., Pernthaler, A., Pernthaler, J., and Herndl, G.J. (2004) Combining catalyzed reporter deposition-fluorescence in situ hybridization and microautoradiography to detect substra-te utilization by Bacteria and Archaea in the deep ocean. Appl. Environ. Microbiol., 70, 4411-4414.

Ushio, M., Makoto, K., Klaminder, J., and Nakano, S.I. (2013) CARD-FISH analysis of prokaryotic community composition and abundance along small-scale vegetation gradients in a dry arctic tundra ecosystem. Soil Biol. Biochem., 64, 147-154.

**Table S1:** *In silico* analysis of the probes. The number of sequences presenting 0-3 mismatches are displayed, with the mismatch location (c = central, t = terminal, (-) = not analysed). For probe mixes, sequences with 1 mismatch were only counted if they had > 0 mismatch with all the probes. Dark background indicates the limit of the probe specificity. (*Italics*) = *unidentified organisms*, [A] Eilers *et al.*, 2001, [B] Not *et al.*, 2002, [C] Not *et al.*, 2004, [D] *This study*.

| | | | **0 mismatch** | | **1 mismatch** | | **2 mismatches** | | **3 mismatches** | |
|---|---|---|---|---|---|---|---|---|---|---|
| **Probe Name** | **Target** | **Database** | **Target** | **Out group** | **Target** | **Outgroup** | **Target** | **Outgroup** | **Target** | **Outgroup** |
| Eub338 | 16S rRNA, *Bacteria* | SSU Ref 114 | 572724 | 2 | $1227_t$ $22352_c$ | $30_c$ | $781_{tc/tt}$ $13979_{cc}$ | $28_{tc}$ $745_{cc}$ | - | - |
| EubII | 16S rRNA, *Bacteria* | SSU Ref 114 | 3699 | 0 | $13_t$ $10011_c$ | 0 | 7082 | $22_c$ | 577259 | 118 |
| EubIII | 16S rRNA, *Bacteria* | SSU Ref 114 | 9915 | 0 | $41_t$ $4287_c$ | $22_c$ | 576984 | 118 | - | - |
| Eub338+EubII+EubIII | 16S rRNA, *Bacteria* | SSU Ref 114 | 586338 | 2 | $1281_t$ $22811_c$ | $30_c$ | - | $28_{tc}$ $745_{cc}$ | - | 211ttc |
| Gam42a | 23S rRNA, *Gammaproteobacteria* | LSU Parc 114 | 6639 | 43 (*83*) | $31_t$ $939_c$ | $1129_c$ (*$4_t$ $334_c$*) | $3_{tt}$ $21_{tc}$ $72_{cc}$ | $44_{tc}$ $143_{cc}$ (*$6_{tc}$ $51_{cc}$*) | $16_{tcc}$ $3_{ccc}$ | $2_{ttc}$ $164_{tcc}$ $480_{ccc}$ (*$26_{tcc}$ $128_{ccc}$*) |
| Gam42a + comp Bet42a | 23S rRNA, *Gammaproteobacteria* | LSU Parc 114 | 6639 | 43 (*83*) | $31_t$ $353_c$ | $102_c$ (*$4_t$ $106_c$*) | - | - | - | - |
| Ros537 [A] | 16S rRNA, *Roseobacter* clade | SSU Ref 114 | 4935 (*1443*) | 51 (*141*) | $29_t$ $296_c$ (*$540_c$*) | $5_t$ $5145_c$ | $1_{tt}$ $91_{tc}$ $88_{cc}$ (*$76_{tc}$ $221_{cc}$*) | $23733_{tc}$ $7142_{cc}$ | - | - |
| Roseo536R | 16S rRNA, *Roseobacter* clade | SSU Ref 114 | 4935 (*1438*) | 51 (*141*) | $29_t$ $296_c$ (*$535_c$*) | $2_t$ $5145_c$ | $1_{tt}$ $7_{tc}$ $172_{cc}$ (*$6_{tc}$ $291_{cc}$*) | $572_{tc}$ $30482_{cc}$ | - | - |
| Roseo536R + comp | 16S rRNA, *Roseobacter* clade | SSU Ref 114 | 4935 (*1438*) | 51 (*141*) | $29_t$ $45_c$ | $2_t$ $15_c$ | - | - | - | - |
| Euk1209 | 18S rRNA, Eukaryotes | SSU Ref 113 | 61525 | 0 | $1272_t$ $4223_c$ | 0 | 794 | 2 | 200 | 74 |
| Chlo01 | 18S rRNA, green lineage | SSU Ref 114 | 3918 | 5396 | 4692 | 46939 | 95 | 7827 | - | - |
| Chlo02 | 18S rRNA, green lineage | SSU Ref 113 | 8099 | 7 | $39_t$ $337_c$ | $3544_c$ | $2_{tt}$ $5_{tc}$ $423_{cc}$ | $88_{tc}$ $21227_{cc}$ | - | - |
| NChlo01 | 18S rRNA, non-Chlorophyta | SSU Ref 113 | 44968 | 111 | $1182_t$ $11169_c$ | $3190_c$ | $159_{tt}$ $843_{tc}$ $2317_{cc}$ | $1_{tt}$ $55_{tc}$ $1388_{cc}$ | - | - |
| Chlo01+NChlo01 [B] | 18S rRNA, Euk. | SSU Ref | 54097 | 3 | $1416_t$ | $272_c$ | - | - | - | - |

| | | | | | | | | | | |
|---|---|---|---|---|---|---|---|---|---|---|
| | phytoplankton | 113 | | | $7117_c$ | | | | | |
| Chlo02+NChlo01 | 18S rRNA, Euk. phytoplankton | SSU Ref 113 | 49132 | 0 | $288_t$ $9805_c$ | $45_c$ | - | - | - | - |
| Euk1209+Chlo02 | 18S rRNA, Euk. phytoplankton | SSU Ref 113 | 61747 | 0 | $1256_t$ $4380_c$ | 0 | - | - | - | - |
| Euk1209+NChlo01 | 18S rRNA, Euk. phytoplankton | SSU Ref 113 | 64956 | 0 | $1181_t$ $3016_c$ | $45_c$ | - | - | - | - |
| Euk1209+NChlo01+ Chlo01 [C] | 18S rRNA, Euk. phytoplankton | SSU Ref 113 | 65588 | 3 | $1198_t$ $2481_c$ | $272_c$ | - | - | - | - |
| Euk1209+NChlo01+ Chlo02 [D] | 18S rRNA, Euk. phytoplankton | SSU Ref 113 | 65065 | 0 | $1273_t$ $3239_c$ | $45_c$ | - | - | - | - |
| Pela01 | 18S rRNA, Pelagophytes | SSU Ref 114 | 42 | 0 | $1_c$ | 0 | $1_{cc}$ | $7_{ct}$ | 0 | $2_{tcc}$ $1006_{ccc}$ |
| Pras04 | 18S rRNA, *Mamiellophyceae* | SSU Ref 114 | 390 | 3 | $1_t$ $17_c$ | $8_t$ $860_c$ | 0 | $2777_{tc}$ $2322_{cc}$ | - | - |

**Table S2:** Strains used in this study, with the corresponding size and culture conditions (n.r. = not required). [a] RCC: Roscoff Culture Collection, France; CCMP: Provasoli-Guillard National Center for Culture of Marine Phytoplankton, USA; DSM: Deusche Sammlung von Mikroorganismen und Zellkulturen GmbH, Germany. [b] 12h:12h cycle.

| **Domain** | **Class** | **Strain [a]** | **Cell size (µm)** | **Culture Medium** | **Light intensity [b] (µE m$^{-2}$ s$^{-1}$)** | **Temperature (°C)** |
|---|---|---|---|---|---|---|
| *Eukarya* | *Prasinophyceae* | *Micromonas sp.* CCMP2099 | 1-3 | L1-Si | 40 | 4 |
| | | *Micromonas pusilla* RCC299 | 2-3 | K | 145 | 20 |
| | *Pelagophyceae* | *Pelagomonas calceolata* CCMP1214, RCC100 | 1-4 | K | 145 | 20 |
| | | *Pelagococcus subviridis* CCMP1429 | 2-4 | L1-Si | 350 | 15 |
| | *Bicosoecid* | *Symbiomonas scintillans* RCC257 | 2-3 | Riz | 4 | 20 |
| | *Chlorarachniophyceae* | *Lotharella reticulosa* RCC375 | 9-16 | K | 100 | 20 |
| *Bacteria* | *Planctomycetacia* | *Planctomyces limnophilus* DSM3776 | 1.1-1.5 | 621 agar | n.r., 500 | 28 |
| | *Verrucomicrobia* | *Verrucomicrobium spinosum* DSM4136 | 0.8-3.8 | 607 agar | n.r., 500 | 28 |
| | *Gammaproteobacteria* | *Halomonas desiderata* DSM9502 | 0.4-2.6 | 762 agar | n.r., 500 | 28 |
| | | *Halomonas muralis* DSM14789 | 0.4-3.0 | 514 agar | n.r., 500 | 28 |
| | *Alphaproteobacteria* | *Roseobacter denitrificans* OCh114, DSM7001 | 0.6-2 | 514 agar | n.r., 500 | 20 |
| | | *Paracoccus aestuarii* B7, DSM19484 | 0.8-2.0 | 545 agar | n.r., 500 | 28 |
| *Archaea* | *Haloarcheon* | *Haloarcheon* msnc14(3) | Pleomorphic 1-3 | SCM liquid | n.r. | 37 |

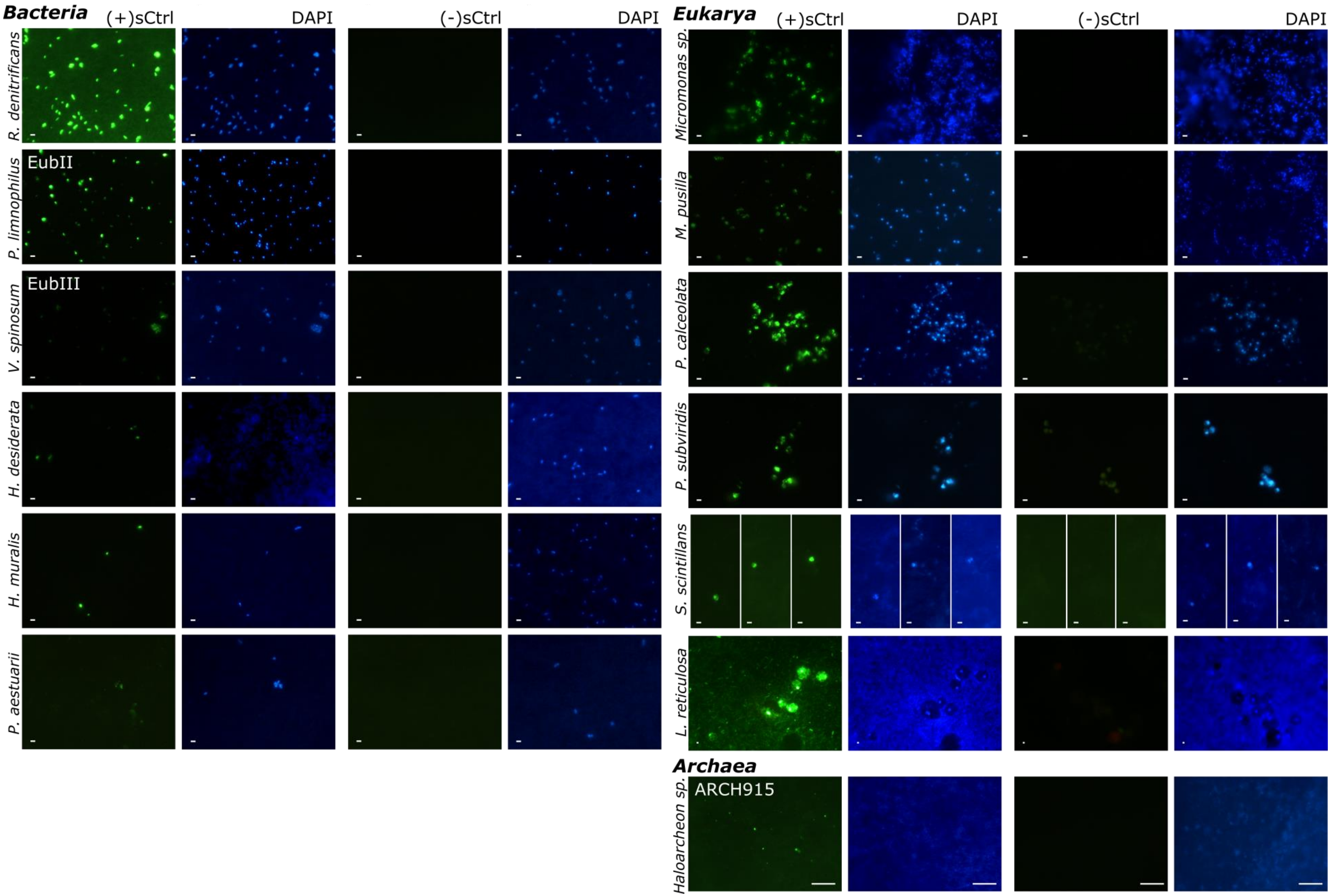

Bacteria
(+)sCtrl
DAPI
(-)sCtrl
DAPI
R. denitrificans
P. limnophilus
EubII
V. spinosum
EubIII
H. desiderata
H. muralis
P. aestuarii
Eukarya
(+)sCtrl
DAPI
(-)sCtrl
DAPI
Micromonas sp.
M. pusilla
P. calceolata
P. subviridis
S. scintillans
L. reticulosa
Archaea
Haloarcheon sp.
ARCH915

-

**Figure S1:** Control hybridizations of the strains. (+)sCtrl : Showing that all strain studied had a potential of positive hybridization with a domain probes from Eubacteria, Eukaryote and Archea, using 50%, 40% and 5% formamide concentration respectively. (-)sCtrl: showing the potential of strains autofluorescence when omitting the probe in hybridization procedure (no probe control).. Scale bars indicate 2 µm.

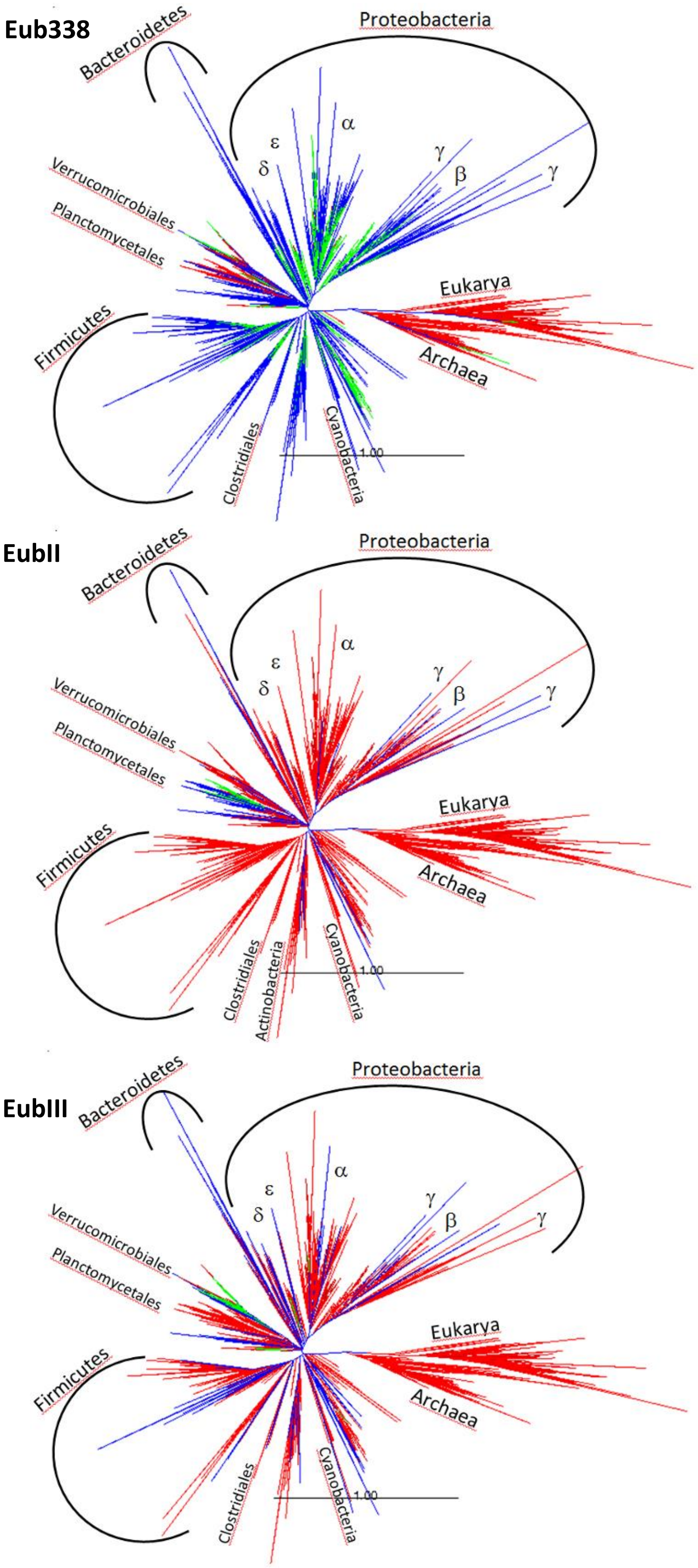


**Figure S2:** Representation of the current Eub338, EubII and EubIII probes specificity onto a phylogenetic tree calculated from the 739,633 DNA sequences encoding the small subunit ribosomal RNA available in the curated Silva SSU ref #111 database. Blue and Green, targets with 0 mismatch. Red, not targeted.

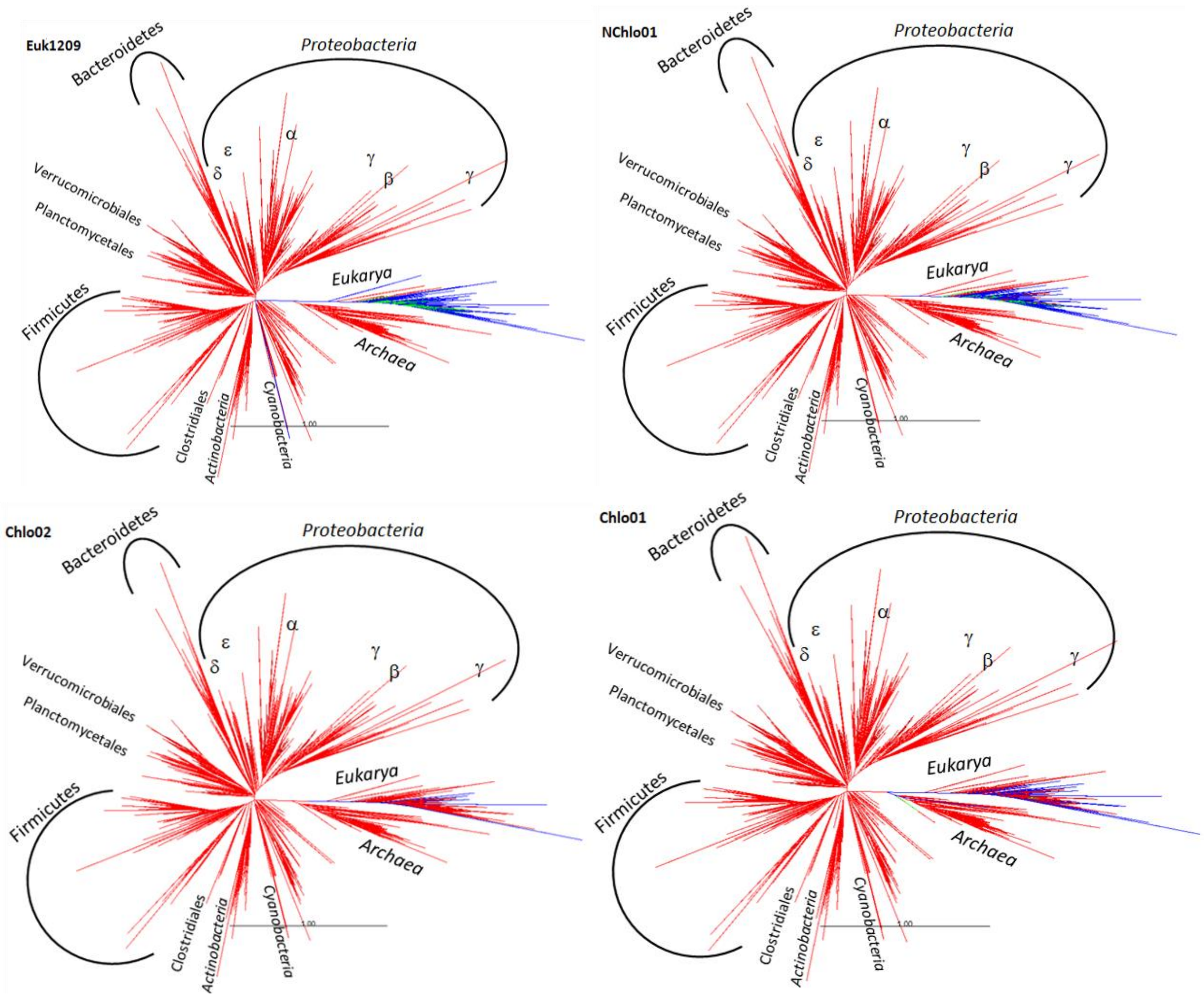


**Figure S3:** Current Euk1209, NChlo01, Chlo02 and Chlo01 probes specificity onto a phylogenetic tree calculated from the 739,633 DNA sequences available in the curated Silva SSU ref #111 database. Blue and Green, targets with 0 mismatch. Red, not targeted.
